# Integrated Computational Modeling Reveals a Structurally Plausible Transient Paclitaxel–NK2R Interaction

**DOI:** 10.3390/bioengineering13080953

**Published:** 2026-08-21

**Authors:** Corina Duda-Seiman, Liliana Mititelu Tartau, Bogdan Hoinoiu, Daniel Pit, Victor Dumitrascu, Alina Doina Tanase, Elena Rusu, Andrei Luca, Eliza Gratiela Popa, Teodora Hoinoiu

**Affiliations:** 1 Titu Maiorescu University, Vacaresti 187, 040051 București, Romania; corina.duda@e-uvt.ro (C.D.-S.); elena.rusu@prof.utm.ro (E.R.); andrei.luca@prof.utm.ro (A.L.); 2 Grigore T. Popa University of Medicine and Pharmacy, 700115 Iasi, Romania; eliza.popa@umfiasi.ro; 3 Department of Oral Rehabilitation and Dental Emergencies, Faculty of Dentistry, “Victor Babes” University of Medicine and Pharmacy, 300041 Timisoara, Romania; hoinoiu@umft.ro; 4 Interdisciplinary Research Center for Dental Medical Research, Lasers and Innovative Technologies, 300041 Timisoara, Romania; 5 Doctoral School, “Victor Babes” University of Medicine and Pharmacy, 300041 Timisoara, Romania; daniel.pit@umft.ro; 6 Center for Advanced Research in Cardiovascular Pathology and Hemostaseology, 300041 Timisoara, Romania; tstoichitoiu@umft.ro; 7 Department I of Nursing, University Clinic of Clinical Skills, “Victor Babes” University of Medicine and Pharmacy, 300041 Timisoara, Romania; 8 Department of Pharmacology, “Victor Babes” University of Medicine and Pharmacy, 300041 Timisoara, Romania; dumitrascu.victor@umft.ro; 9 Department of Professional Legislation in Dental Medicine, Faculty of Dental Medicine, “Victor Babes” University of Medicine and Pharmacy, 300041 Timisoara, Romania; tanase.alina@umft.ro

**Keywords:** paclitaxel, NK2R, GPCR, molecular docking, molecular dynamics, computational pharmacology, breast cancer, drug–receptor interaction, in silico modeling

## Abstract

Background: Paclitaxel is a cornerstone chemotherapeutic agent widely used in breast cancer treatment, primarily through the stabilization of microtubule dynamics. Beyond its canonical tubulin-targeting activity, increasing evidence suggests that paclitaxel may engage additional molecular targets, contributing to its complex pharmacological profile. In this study, an integrated computational workflow was applied to evaluate the structural compatibility between paclitaxel and the neurokinin-2 receptor (NK2R), a G protein-coupled receptor involved in tumor-associated inflammatory and proliferative signaling pathways. Physicochemical profiling and target prediction were performed using SwissADME and SwissTargetPrediction, followed by molecular docking and molecular dynamics simulations using AutoDock Vina and GROMACS 2024.1. Paclitaxel exhibited physicochemical properties consistent with transient interactions in hydrophobic transmembrane environments. Docking analysis identified a plausible binding mode within the NK2R transmembrane cavity, primarily stabilized by hydrophobic contacts. Molecular dynamics simulations over 100 ns revealed stable ligand occupancy and overall complex stability, while MM-PBSA calculations indicated a favorable transient association. The predicted interaction is consistent with secondary or non-canonical receptor engagement. While NK2R is not established as a pharmacological target of paclitaxel, the results support the structural feasibility of a previously uncharacterized receptor interaction and provide a reproducible computational framework for exploring GPCR-associated effects of cytotoxic agents.

## 1. Introduction

Paclitaxel is a diterpenoid antineoplastic agent widely used in the treatment of breast, ovarian, and non-small cell lung cancers due to its ability to stabilize microtubules and inhibit mitotic progression [1,2]. In clinical practice, albumin-bound paclitaxel formulations such as Abraxane^®^ have improved drug solubility and reduced the toxicity associated with solvent-based formulations [3]. Despite its established mechanism of action involving β-tubulin stabilization, accumulating evidence indicates that paclitaxel may also exert additional biological effects through interactions with alternative cellular targets and signaling pathways [4,5].

The identification of off-target interactions has become increasingly relevant in cancer pharmacology, as such interactions may contribute to therapeutic efficacy, resistance mechanisms, or adverse effects [6]. Computational approaches integrating ligand-based target prediction, structural bioinformatics, molecular docking, and molecular dynamics simulations are widely used to explore potential receptor–ligand interactions and generate mechanistic hypotheses for experimentally uncharacterized drug activities [7,8,9]. These methods do not establish biological activity on their own but can highlight structurally plausible interactions suitable for further experimental evaluation.

G protein-coupled receptors (GPCRs) represent one of the largest and most pharmacologically relevant receptor families, with roles in tumor proliferation, angiogenesis, migration, inflammation, and treatment response [10,11]. Neurokinin receptor 2 (NK2R), also known as tachykinin receptor 2, is a class A GPCR activated primarily by neurokinin A and implicated in cancer-related signaling pathways [12,13]. Tachykinin signaling has been associated with breast cancer progression through modulation of inflammatory and proliferative processes [14,15]. Recent availability of high-resolution cryo-electron microscopy structures of NK2R has enabled detailed computational characterization of ligand-accessible binding environments [16].

Paclitaxel is an amphipathic molecule containing both hydrophobic aromatic regions and polar functional groups, a property compatible with transient accommodation in hydrophobic transmembrane cavities [17]. Although it is structurally distinct from classical neurokinin ligands, ligand-based target prediction platforms ranked NK2R among the top predicted GPCR-associated targets [18]. This observation motivated a focused structural assessment of potential compatibility between paclitaxel and the NK2R binding site.

Therefore, this study investigated the potential interaction between paclitaxel and NK2R using an integrated in silico workflow including ADMET profiling, target prediction, receptor structural characterization, molecular docking, and molecular dynamics simulations. The aim was not to characterize NK2R as a pharmacological target of paclitaxel, but to assess whether computational evidence supports a structurally plausible secondary interaction that could justify future experimental validation.

## 2. Materials and Methods

### 2.1. Computational Strategy and Rationale

An integrated in silico workflow was used to explore the potential interaction between paclitaxel and NK2R. Physicochemical and ADMET properties of paclitaxel were evaluated using SwissADME, followed by ligand-based target prediction with SwissTargetPrediction. Structural and physicochemical features of NK2R were analyzed using UniProt, ProtParam, and ProtScale [19,20,21,22]. The experimentally determined NK2R structure (PDB ID: 7XWO) was prepared for molecular visualization and docking analysis. Molecular docking and molecular dynamics simulations were subsequently performed to evaluate the structural compatibility and theoretical stability of the proposed NK2R–paclitaxel complex. Because the study was designed as a hypothesis-generating computational investigation, all findings were interpreted qualitatively and not as evidence of biological activity.

### 2.2. Integrated Computational Workflow

The computational pipeline consisted of three primary stages:

- Ligand-level analysis (SwissADME)

Physicochemical descriptors (MW, logP, TPSA, hydrogen bonding, rotatable bonds) and pharmacokinetic parameters (GI absorption, BBB permeability, P-gp substrate status, CYP inhibition) were calculated for paclitaxel using SwissADME, a free web-based tool for ADME and drug-likeness prediction developed by the Swiss Institute of Bioinformatics [23]. These parameters established the baseline molecular profile and were subsequently used as inputs to predict the potential protein targets compatible with the ligand’s amphipathic structure.

- Target-level prediction (SwissTargetPrediction + UniProt)

It should be noted that SwissTargetPrediction was used exclusively for 2D chemical similarity-based target prediction and was not employed for generating three-dimensional ligand coordinates used in docking simulations.

The SMILES string of paclitaxel (PubChem CID: 36314) was retrieved from PubChem and submitted to the SwissTargetPrediction software (2019 version) for 2D similarity-based target prediction [24]. This tool ranks potential human protein targets based on structural similarities and bioactivity data from known ligands [25]. The 15 predicted proteins were retrieved and categorized by functional class (GPCRs, kinases, enzymes, etc.) using UniProt annotations [26]. To ensure objectivity, the selection of the ranked target was based on the following a priori criteria:

- Probability score ≥ 0.20;
- Known relevance of the protein in breast cancer biology (from literature);
- Potential for hydrophobic or amphipathic ligand binding.

NK2R was prioritized a priori based on predefined criteria including SwissTargetPrediction probability, documented involvement in breast cancer biology, and structural compatibility with amphipathic ligands. Docking and molecular dynamics simulations were performed only after this unbiased target-selection procedure.

- Receptor-level characterization (ProtParam, ProtScale, UCSF Chimera)

The structural and physicochemical properties of NK2R (UniProt ID: P21452) were characterized using ProtParam and ProtScale to determine hydrophobicity, aliphatic index, and surface flexibility profiles. The experimentally determined 3D structure (PDB ID: 7XWO) was visualized in UCSF Chimera to examine electrostatic and hydrophobic domains that are potentially compatible with the amphipathic features of paclitaxel [27].

The experimentally determined three-dimensional structure (PDB ID: 7XWO), resolved by single-particle cryo-electron microscopy, represents human NK2R in complex with a peptide agonist and associated G-protein components. Because no experimentally determined apo human NK2R structure was available, this structure was selected as the best available template. Prior to structural analysis and docking procedures, all non-receptor components were removed, and only the receptor coordinates were retained for further computational preparation. The use of an active-state receptor conformation is acknowledged as a structural limitation and is discussed accordingly in Section 5.

### 2.3. Conceptual Framework for Interpreting Paclitaxel’s Molecular Behavior

Although paclitaxel is clinically delivered via albumin-bound nanoparticles, its pharmacological behavior is primarily determined by the drug’s molecular properties, which dictate potential receptor interactions after release. Consequently, paclitaxel was modeled as the ligand of interest, while nanoparticle-specific features, such as size, polarity, and transport mechanisms, were considered qualitatively when interpreting the pharmacokinetic and pharmacodynamic outcomes [11,12,28,29,30,31]. This strategy follows established precedents in nanomedicine modeling, where intrinsic drug descriptors are evaluated computationally, and nanoparticle-related parameters are addressed in the discussion.

### 2.4. Target Selection and Comparative Context

NK2R was selected based on a systematic evaluation of all predicted GPCRs with probability scores ≥ 0.20, considering their biological relevance to breast cancer using UniProt and PubMed data. Among these candidates, NK2R has been reported to be associated with tumor proliferation, angiogenesis, and inflammatory signaling pathways [17,18,32,33,34]. The transmembrane region of NK2R also exhibits a highly hydrophobic topology, compatible with amphipathic ligands such as paclitaxel. These factors support prioritizing NK2R as the primary receptor for subsequent structural analyses.

### 2.5. Molecular Docking and Molecular Dynamics Analysis

#### 2.5.1. Molecular Docking

Molecular docking was performed to predict the binding pose of paclitaxel within the NK2R structure and to estimate the corresponding docking score. Docking was performed using AutoDock Vina v1.2.3 within the PyRx 0.9.8 interface, employing the experimentally determined NK2R structure (PDB ID: 7XWO), resolved by single-particle cryo-electron microscopy [35], protein preparation involved removing the co-crystallized peptide ligand. PDB ID 7XWO corresponds to an engineered human NK2R signaling complex composed of multiple protein entities, including the receptor chain (chain B), a cytochrome b562 fusion partner, a peptide agonist, and G-protein α, β1, and γ subunits. For docking preparation, only the NK2R receptor chain (chain B) was retained. All other protein chains, including G-protein subunits and fusion partners, as well as the co-determined peptide agonist, were removed prior to structural preparation.

After isolating the NK2R receptor chain, non-receptor protein components were removed. The resulting structure was then prepared for docking by adding polar hydrogens and assigning Gasteiger charges using AutoDockTools version 1.5.7. The receptor was treated as an isolated protein model throughout the docking calculations. This simplification was intentionally adopted to evaluate structural compatibility between paclitaxel and the receptor binding cavity while minimizing additional system complexity.

Because the present study was designed to explore the structural plausibility of a previously uncharacterized interaction rather than to establish agonist-like receptor activation, the active-state structure was used as a defined structural template. Nevertheless, potential state-dependent differences in paclitaxel recognition cannot be excluded and were not directly assessed in the present study. The selected structure provides a well-defined transmembrane cavity suitable for evaluating potential hydrophobic ligand accommodation. Nevertheless, we acknowledge that conformational differences between active and inactive states may influence pocket geometry and ligand accessibility. Polar hydrogen atoms were added, and Gasteiger charges were assigned using AutoDock Tools. The receptor was treated as rigid during docking. Paclitaxel (PubChem CID: 36314) [24] was geometry-optimized using the MMFF94 force field and converted to PDBQT format using Open Babel v3.1.1. The canonical SMILES string of paclitaxel (PubChem CID: 36314) was retrieved from PubChem. Three-dimensional coordinates were generated using OpenBabel (v3.1.1) with explicit stereochemical constraints preserved according to the experimentally established Taxol stereoisomer. Initial 3D structure generation was followed by a conformational search using a weighted rotor search algorithm, after which the lowest-energy conformer was subjected to MMFF94 force-field optimization to relieve steric strain while maintaining all 11 chiral centers in their native configuration. Ring systems were treated with fixed stereochemistry to prevent artificial inversion. The resulting lowest-energy conformer was exported in PDB format and subsequently converted to PDBQT using AutoDockTools, with appropriate assignment of rotatable bonds. All stereogenic centers were preserved in their native configuration throughout file conversion and preparation steps. Given the structural complexity of paclitaxel, including its polycyclic taxane core and multiple fused ring systems, geometry optimization was performed using OpenBabel under the MMFF94 force field. This step was applied to relieve minor steric strain while maintaining experimentally determined stereochemistry and ring topology. No chiral inversions or artificial conformational distortions were introduced. The final PDBQT file used for docking was generated using AutoDockTools, with rotatable bonds assigned only to appropriate side chains while preserving the rigid polycyclic core. The top- ranked pose (docking score = −8.1 kcal mol^−1^) was selected for subsequent MD analysis. The docking search space was defined to encompass the experimentally characterized orthosteric binding cavity of NK2R. The grid box was positioned to include the entire canonical neurokinin-binding pocket while providing sufficient space to accommodate the relatively large paclitaxel molecule during conformational sampling.

The ligand was treated as flexible, allowing rotation of all non-ring rotatable bonds. The docking grid box was manually defined in PyRx based on the experimentally characterized orthosteric binding cavity of NK2R identified in the cryo-EM structure. The search space was centered on the canonical neurokinin-binding pocket, encompassing residues 240–270, with coordinates (x = 42.5, y = −17.3, z = 9.6), a grid size of 30 × 30 × 30 Å, and an exhaustiveness value of 16. The grid dimensions were selected to fully cover the orthosteric cavity while allowing sufficient space for conformational sampling of the relatively large paclitaxel molecule.

The initial three-dimensional structure of paclitaxel was retrieved from the PubChem database (CID: 36314) as the precomputed 3D conformer available in SDF format. PubChem provides standardized computational conformers generated using standardized geometry optimization protocols.

The subsequent ligand preparation procedure was performed as follows: the SDF file was converted to PDB format using Open Babel (v3.1.1), followed by local geometry optimization under the MMFF94 force field to relieve minor steric strain while preserving all 11 experimentally established stereogenic centers and the rigid taxane ring system. The optimized structure was then converted to PDBQT format using AutoDockTools, where Gasteiger charges were assigned and rotatable bonds were defined.

The paclitaxel structure used in the present study corresponds to the experimentally established natural Taxol stereoisomer, representing the clinically active form of paclitaxel. No epimeric forms or alternative stereoisomers were generated or included in the docking or molecular dynamics simulations.

RMSD values were calculated for the protein backbone atoms and for paclitaxel heavy atoms relative to the initial docking conformation. For each system, three independent 100 ns simulations were performed, and the RMSD profiles represent the averaged trajectories obtained from these runs.

Because the available NK2R structure is bound to a peptide agonist rather than a comparable small-molecule ligand, the docking protocol was used for structural compatibility assessment rather than redocking-based validation.

#### 2.5.2. Molecular Dynamics (MD) Simulations and Trajectory Analysis

All-atom MD simulations were performed on the NK2R–paclitaxel complex to assess the structural stability and dynamic behavior of the docked system. Production MD simulations were performed for 100 ns, with trajectory frames saved every 0.5 ns for subsequent analyses. Before the production run, the system was energy-minimized and equilibrated under constant temperature (310 K) and pressure (1 bar). Temperature and pressure were regulated using standard coupling algorithms implemented in GROMACS. Molecular dynamics simulations were performed using GROMACS 2024.1 in combination with the CHARMM36 force field [36].

The system was solvated, neutralized with counterions, and supplemented with NaCl to a final concentration of 0.15 M. The MMFF94 force field was used solely for the preliminary geometry optimization of paclitaxel prior to docking and was not employed during the molecular dynamics simulations. For MD, all ligand parameters were generated using the CHARMM General Force Field (CGenFF), ensuring full compatibility with the CHARMM36 force field applied to the protein. Because the objective of the present work was to evaluate the intrinsic stability of the predicted receptor–ligand complex in a hypothesis-generating framework, molecular dynamics simulations were performed in explicit aqueous solvent without an explicit lipid bilayer.

The trajectories were analyzed for structural stability (RMSD, RMSF, and radius of gyration) and hydrogen-bond interactions.

The equilibrated portions of the trajectories were subsequently used for MM-PBSA binding free energy analysis.

#### 2.5.3. MM-PBSA Binding Free Energy Calculations

Binding free energy calculations were performed using a custom Python 3.11 script developed in-house, which implements the standard MM-PBSA formalism applied to GROMACS trajectory files. A total of 200 evenly spaced frames were extracted from the equilibrated portion of each trajectory (80–100 ns interval), as determined from RMSD convergence analysis. Molecular mechanics energy components, including van der Waals and electrostatic contributions, were calculated using the CHARMM36 force field parameters consistent with the MD simulations. Polar solvation energies were computed using the Poisson–Boltzmann (PB) model with a solvent dielectric constant of 80 and a solute dielectric constant of 2, while non-polar solvation energies were estimated using a solvent-accessible surface area (SASA)-based approach.

The binding free energy (ΔG_bind) was defined according to the general MM-PBSA formulation:

ΔG_bind = ΔE_MM + ΔG_solv − TΔS

where ΔE_MM includes electrostatic and van der Waals terms, and ΔG_solv includes polar and non-polar solvation contributions. In the present calculations, the entropic term (TΔS) was not included; therefore, the reported MM-PBSA values represent estimates based on molecular-mechanics and solvation terms rather than fully entropy-corrected absolute binding free energies. Therefore, the resulting MM-PBSA values should be interpreted as estimates based on molecular-mechanics and solvation terms rather than as fully entropy-corrected absolute binding free energies. For a large and conformationally complex ligand such as paclitaxel, omission of an unfavorable binding entropy contribution could result in a more favorable (more negative) MM-PBSA energetic estimate. The reported MM-PBSA values represent the mean ± standard deviation obtained from three independent 100 ns trajectories.

## 3. Results

All computational outputs were interpreted to provide analytical depth rather than being presented as raw data. The quantitative results from SwissADME and SwissTargetPrediction were reformatted, summarized, and discussed in the context of the known pharmacokinetic and pharmacodynamic properties of paclitaxel.

### 3.1. Physicochemical Characterization of Paclitaxel

The chemical and physicochemical properties of paclitaxel, the active component of Abraxane, were obtained from the PubChem database (CID: 36314) [24]. Paclitaxel, the active compound of Abraxane, has a MW of 853.9 g/mol, a calculated logP (logarithm of the partition coefficient) of 2.5, four hydrogen bond donors (HBD), 14 hydrogen bond acceptors (HBA), 14 rotatable bonds, and a topological polar surface area (TPSA) of 221 Å^2^. These values indicate high polarity and limited passive permeability, consistent with intravenous administration and albumin-mediated transport. The quantitative molecular descriptors are listed in Table 1.

### 3.2. Pharmacokinetic and Drug-Likeness Predictions

SwissADME modeling predicted low GI absorption and no BBB permeability. The molecule was identified as a P-gp substrate but not an inhibitor of major cytochrome P450 (CYP) isoenzymes. The predictions are quantitatively summarized in Table 2.

The physicochemical descriptors of paclitaxel, including its high molecular weight, polar surface area of 221 Å^2^, and logP of 2.5, directly influence its interaction profile with hydrophobic receptor environments such as NK2R. Its amphipathic structure, containing both hydrophilic ester and hydrophobic aromatic domains, supports partial membrane penetration and transient accommodation within the lipophilic GPCR pockets. Limited gastrointestinal absorption and lack of blood–brain barrier permeability are consistent with intravenous administration and local tumor accumulation. Collectively, these ADMET and drug-likeness properties help explain paclitaxel’s predicted preference for transmembrane, lipid-accessible binding cavities rather than soluble cytosolic proteins, providing a mechanistic link between ligand physicochemistry and computationally predicted receptor-binding behavior.

According to the Lipinski, Ghose, Veber, Egan, and Muegge criteria, paclitaxel does not meet the typical parameters of an orally bioavailable drug candidate. Specifically, it violates Lipinski’s rules due to excessive molecular weight and a high number of hydrogen bond acceptors; the Ghose filter indicates suboptimal logP and molar refractivity, reflecting a poor balance between hydrophilicity and lipophilicity; the Veber rule flags the compound for too many rotatable bonds and a large TPSA, potentially hindering membrane permeability; the Egan rule indicates excessive polarity; and the Muegge filter places paclitaxel outside the optimal chemical space for drug-like molecules. These violations collectively resulted in a low predicted oral bioavailability score (0.17), suggesting inefficient gastrointestinal tract absorption.

It should be noted that violations of classical drug-likeness rules do not preclude clinical efficacy. Numerous approved natural products and anticancer agents, including paclitaxel, exceed Lipinski’s criteria because of their structural complexity, yet remain highly effective through alternative administration routes and advanced formulation strategies [37,38]. In particular, intravenous delivery and nanoparticle-based formulations, such as albumin-bound paclitaxel (Abraxane^®^), overcome the limitations associated with poor oral bioavailability and unfavorable physicochemical properties [39,40]. These considerations emphasize that classical drug-likeness rules should be interpreted within the context of the intended route of administration and formulation design rather than as absolute indicators of therapeutic potential.

These findings highlight that the pharmacological efficacy of paclitaxel relies primarily on carrier-mediated transport rather than passive diffusion. Although the compound does not conform to the classical small-molecule drug-like criteria, this behavior is consistent with macromolecular or nanoparticle-based delivery systems, in which therapeutic performance depends on the carrier properties rather than conventional molecular descriptors. The low predicted oral bioavailability and minimal CYP inhibition are consistent with the known pharmacokinetic profile of intravenously administered paclitaxel, which exhibits limited metabolic interactions.

### 3.3. Target Prediction and Functional Classification

SwissTargetPrediction analysis identified several potential protein classes that are potentially compatible with paclitaxel, including GPCRs, kinases, enzymes, and cytosolic proteins. Among the predicted GPCR-associated targets, NK2R showed one of the highest probability scores (0.32), followed by dopamine D2 and β-adrenergic receptors. Although a SwissTargetPrediction probability score of 0.32 does not constitute evidence of receptor binding or pharmacological activity, it indicates a relatively high degree of structural similarity between paclitaxel and known ligands associated with NK2R within the reference dataset. In the context of this study, the score served as a prioritization criterion for selecting NK2R for subsequent structural analyses, including molecular docking and molecular dynamics simulations, rather than as proof of biological interaction.

**Figure 1:**
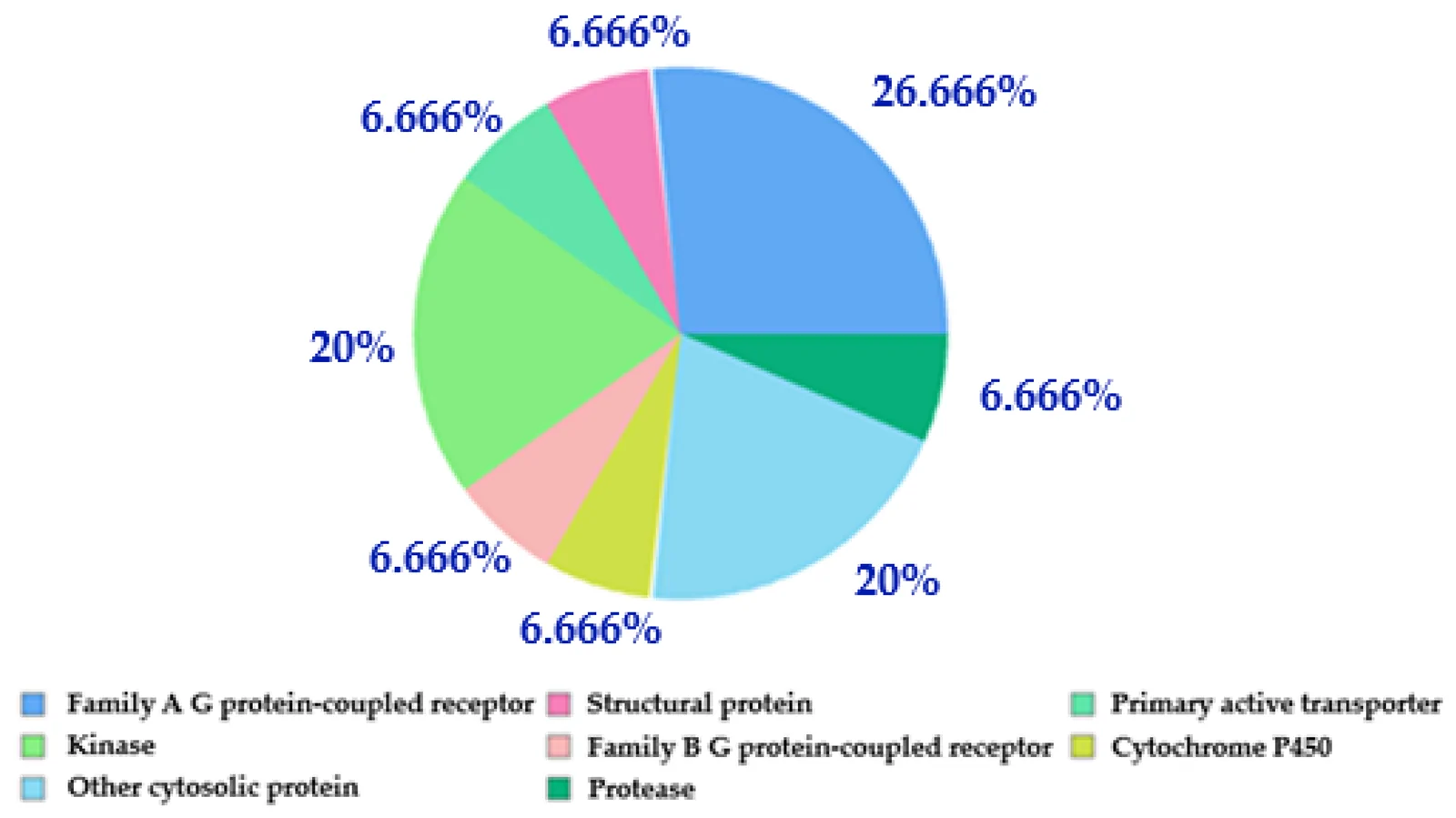
Predicted target families of paclitaxel obtained using SwissTargetPrediction.

Figure 1 summarizes the distribution of the predicted target families obtained using SwissTargetPrediction, with GPCRs representing the largest functional category. Because GPCRs are frequently involved in tumor-associated signaling pathways, this receptor class was selected for further structural evaluation.

NK2R (tachykinin receptor 2) was considered a suitable candidate for additional analysis because previous studies have associated tachykinin signaling with tumor proliferation, angiogenesis, inflammatory responses, and breast cancer progression [17,18,41,42,43]. In addition, the receptor possesses a predominantly hydrophobic transmembrane binding environment compatible with amphipathic ligands.

To provide comparative context for the target prediction workflow, several previously reported NK2R ligands from BindingDB were analyzed using the same prediction platform. These reference compounds showed high predicted interaction probabilities, indicating that the platform can recognize structural features associated with established NK2R-binding chemotypes. However, because paclitaxel is structurally distinct from classical neurokinin ligands, the observed probability score should be considered in context and does not by itself indicate receptor specificity Owing to its large, highly substituted scaffold and substantial structural divergence from canonical neurokinin ligands, the probability score of 0.32 was used solely as an initial target-prioritization criterion rather than as evidence for target assignment. The proposed interaction was subsequently evaluated using complementary structure-based approaches, including molecular docking, molecular dynamics simulations, and MM-PBSA binding free-energy calculations.

Functional annotation obtained from UniProt identified NK2R (UniProt ID: P21452) as a class A multipass transmembrane GPCR involved in neuropeptide-mediated signaling and inflammatory regulation. Comparative analysis of the predicted GPCR-associated targets suggested that NK2R combines computational compatibility with potential biological relevance in cancer-associated signaling pathways, providing a rationale for subsequent structural and molecular dynamics analyses.

### 3.4. Functional and Structural Characterization of NK2R

Among the predicted targets obtained from SwissTargetPrediction, NK2R showed the highest probability of interaction with paclitaxel (score = 0.32), followed by other GPCRs, such as dopamine D2 (0.21) and β-adrenergic receptors (0.19). This relatively high probability value supports a nonrandom theoretical similarity between paclitaxel and known NK2R ligands.

Functional annotation in UniProt showed that NK2R (UniProt ID: P21452) is a multi-pass transmembrane GPCR involved in signal transduction, smooth muscle regulation, and inflammatory responses. These functions are relevant to tumor proliferation and angiogenesis in breast cancer. A comparative analysis of the predicted GPCRs indicated that NK2R has the strongest reported association with tumor biology, providing a rationale for its selection as the top-ranked receptor for structural evaluation.

UniProt annotation describes NK2R (UniProt ID: P21452) as a 398-amino acid multipass transmembrane GPCR with an MW of 44.4 kDa. Physicochemical profiling using ProtParam and ProtScale yielded an aliphatic index of 98.34 and a positive GRAVY score of +0.47, confirming a predominantly hydrophobic core, suitable for lipophilic ligand binding. The theoretical isoelectric point (pI = 8.85) indicates a slightly basic protein surface. Rather than providing a residue-by-residue analysis, the data emphasize the overall amphipathic topology of the receptor. It consists of a hydrophobic transmembrane domain surrounded by hydrophilic extracellular loops, which is consistent with ligand accessibility at the receptor surface. Comparative BLASTp (version 2.15.0) analysis indicated > 98% sequence identity between human NK2R and its orthologs in *Pan troglodytes* and *Macaca mulatta*, confirming their high evolutionary conservationamong the primate species examined.

Hydrophobicity profiles generated using the Kyte–Doolittle and Hopp–Woods scales suggested alternating hydrophobic and hydrophilic segments corresponding to the transmembrane and extracellular domains of the receptors.

**Figure 2:**
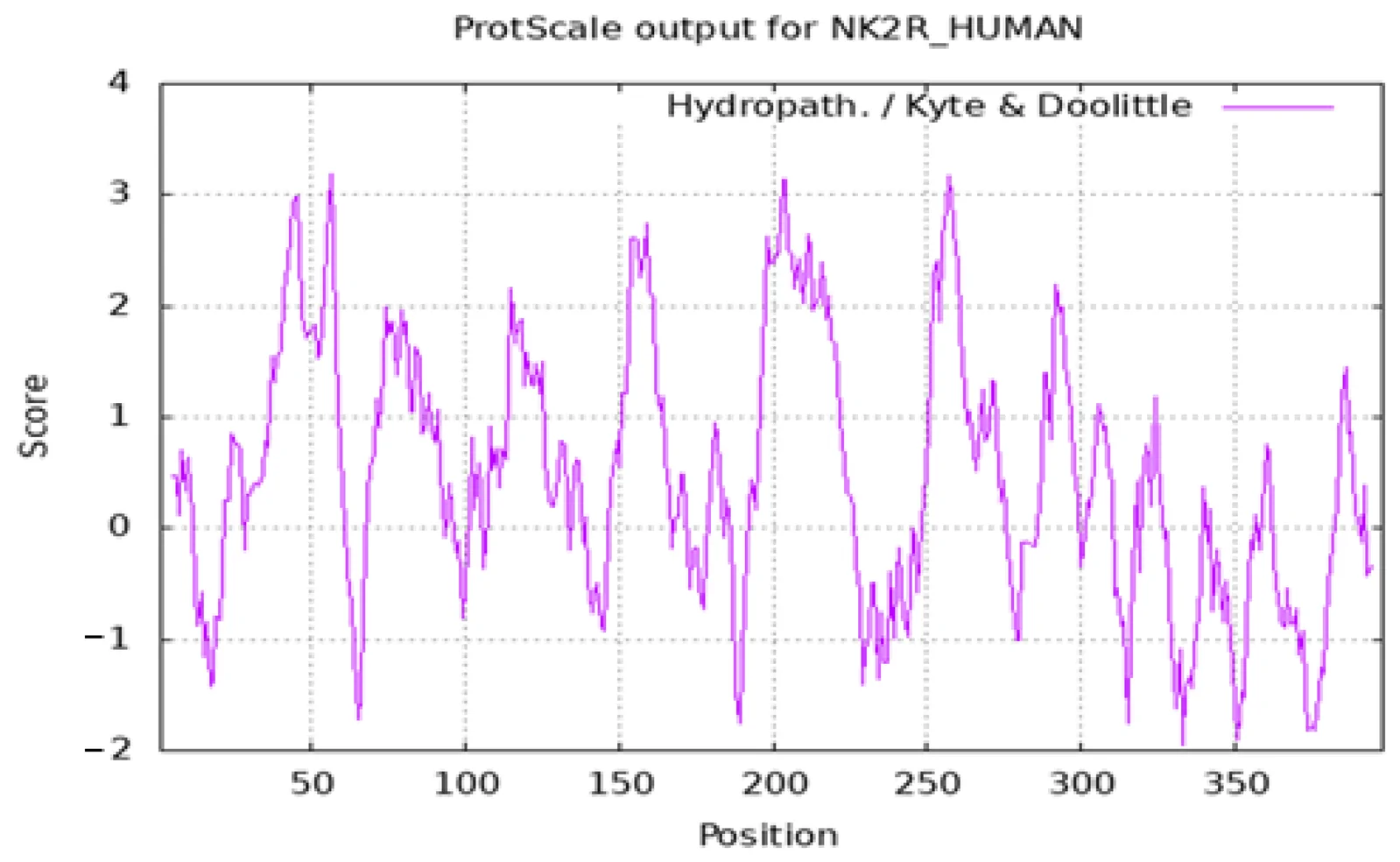
Hydrophobicity distribution along the NK2R sequence (Kyte–Doolittle scale).

The Kyte–Doolittle scale analysis revealed that the protein’s strongest hydrophilic region (−1.944) was at amino acid 333, whereas the most hydrophobic region (+2.989) was at amino acid 258 (Figure 2). These findings suggest that the protein is hydrophobic.

**Figure 3:**
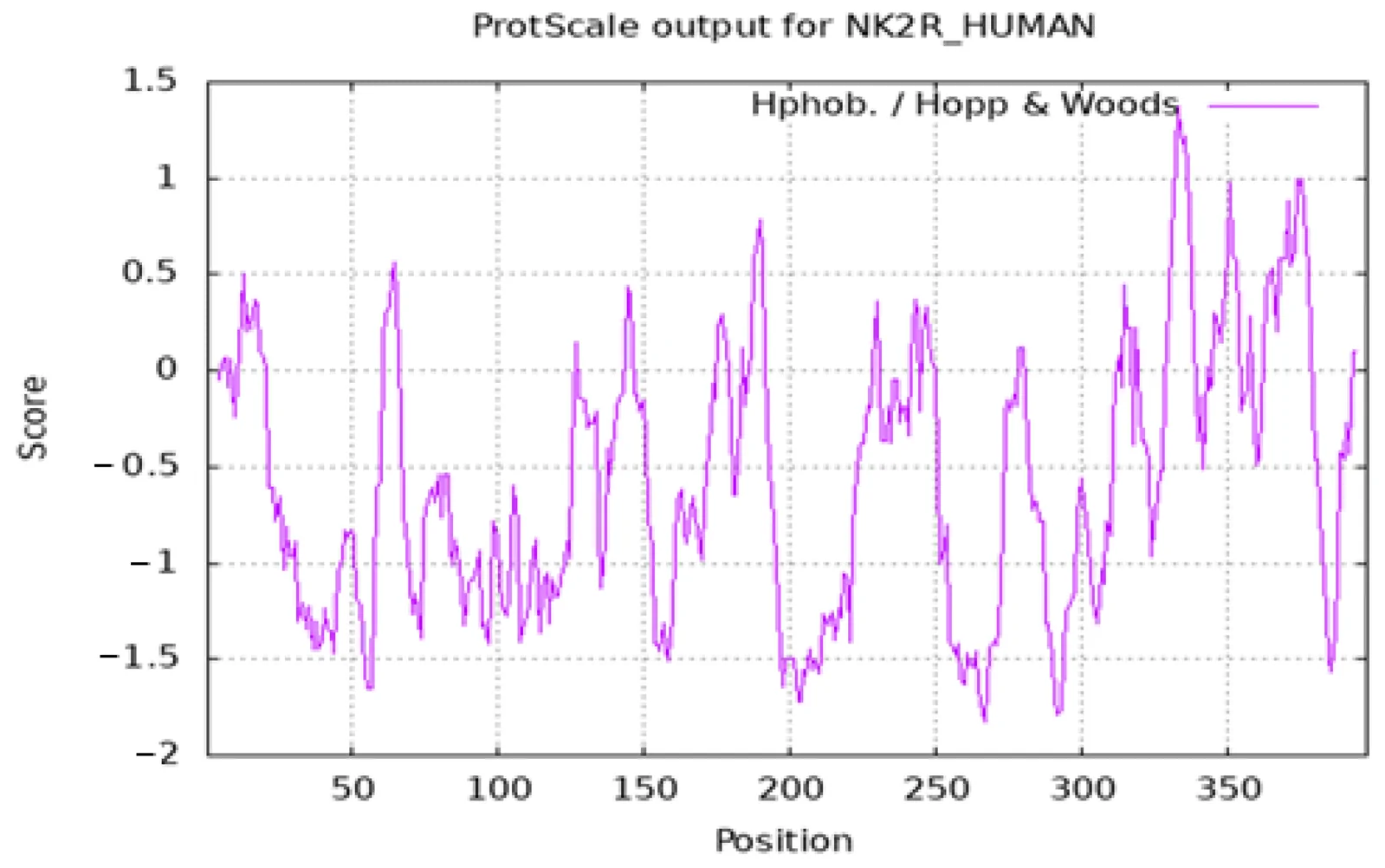
Hydrophobicity profile of the NK2R sequence according to the Hopp–Woods scale.

For the Hopp–Woods scale, the most hydrophobic region of the protein chain had a value of −1.822 at amino acid residue 267. The most hydrophilic region had a value of +1.378 for residue 333 (Figure 3). These values indicate that the protein exhibits a predominantly hydrophobic character.

ProtParam analysis revealed that NK2R has a theoretical isoelectric point (pI) of 8.85. The instability index was calculated to be 40.16, suggesting that the protein is marginally unstable under in vitro conditions. The aliphatic index (98.34) indicates a high proportion of aliphatic side chains, conferring thermostability, and a GRAVY score (0.473) reflected an overall hydrophobic nature consistent with its transmembrane localization.

ProtScale analysis revealed alternating hydrophobic and hydrophilic regions along the polypeptide chain, corresponding to the transmembrane helices and extracellular loops, respectively. The most hydrophobic segments are between residues 240–270, while the most hydrophilic regions were near residues 320–340. The average flexibility score (0.46) indicates moderate flexibility, consistent with the receptor’s conformational adaptability during ligand binding and signal transduction.

**Figure 4:**
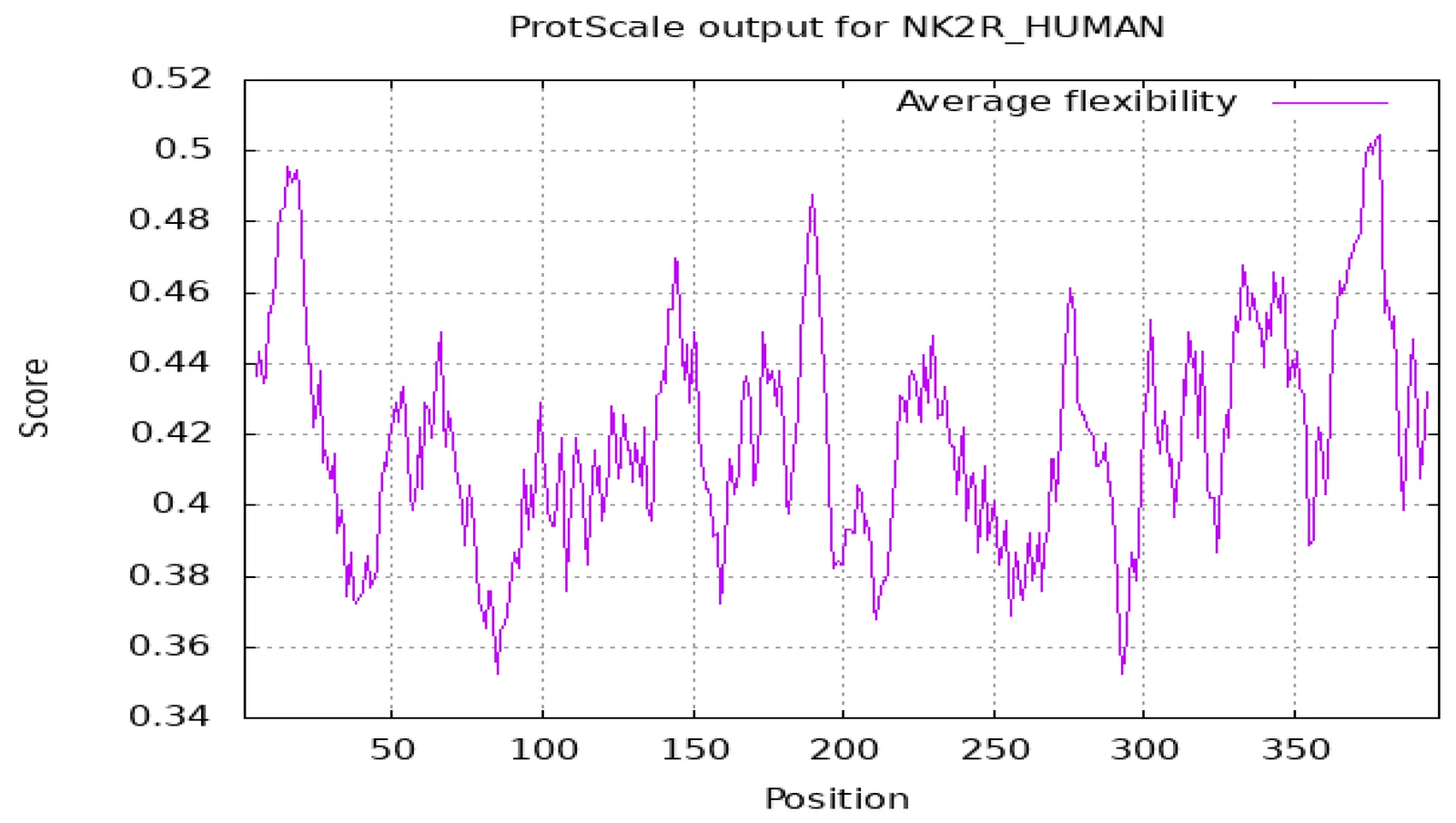
Hydrophobicity distribution graph using the average flexibility scale for the entire protein chain of NK2R.

Figure 4 shows the mean flexibility along the NK2R protein chain. The most flexible region of the protein (0.504) occurs at residue 378, whereas its stiffest region (0.371) is at residue 210, suggesting that the protein is relatively inflexible (Figure 4).

### 3.5. Structural Visualization of NK2R

To prevent structural misinterpretation arising from the multi-component nature of PDB 7XWO, all structural visualizations presented in this study were generated using the isolated NK2R receptor chain (chain B) after removal of fusion partners and G-protein subunits. The β1 subunit of the heterotrimeric G protein (chain C) was not included in surface mapping or electrostatic potential analyses.

**Figure 5:**
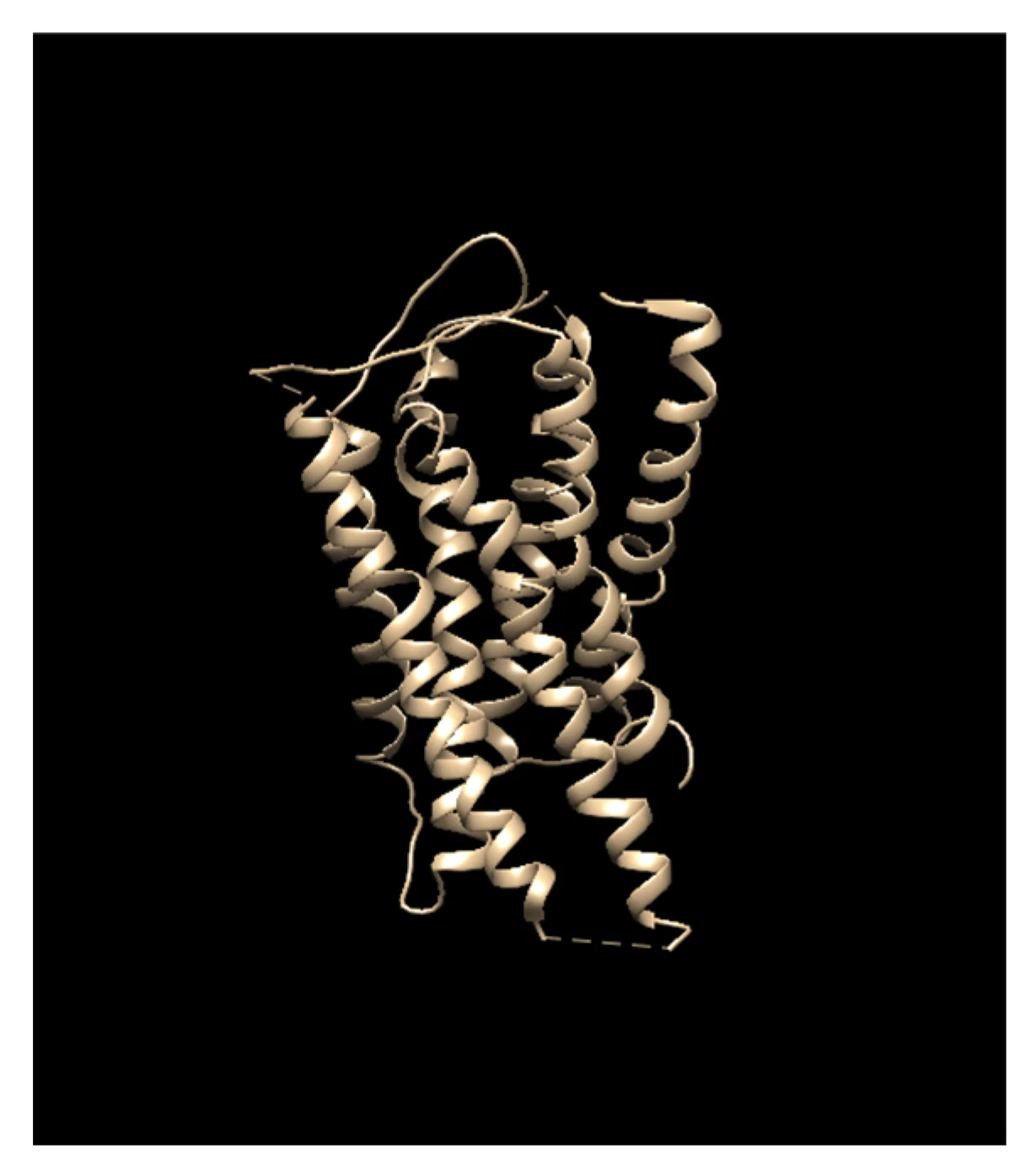
Three-dimensional structure of human NK2R derived from PDB ID: 7XWO after removal of co-crystallized peptide ligand and G-protein components, visualized using UCSF Chimera.

**Figure 6:**
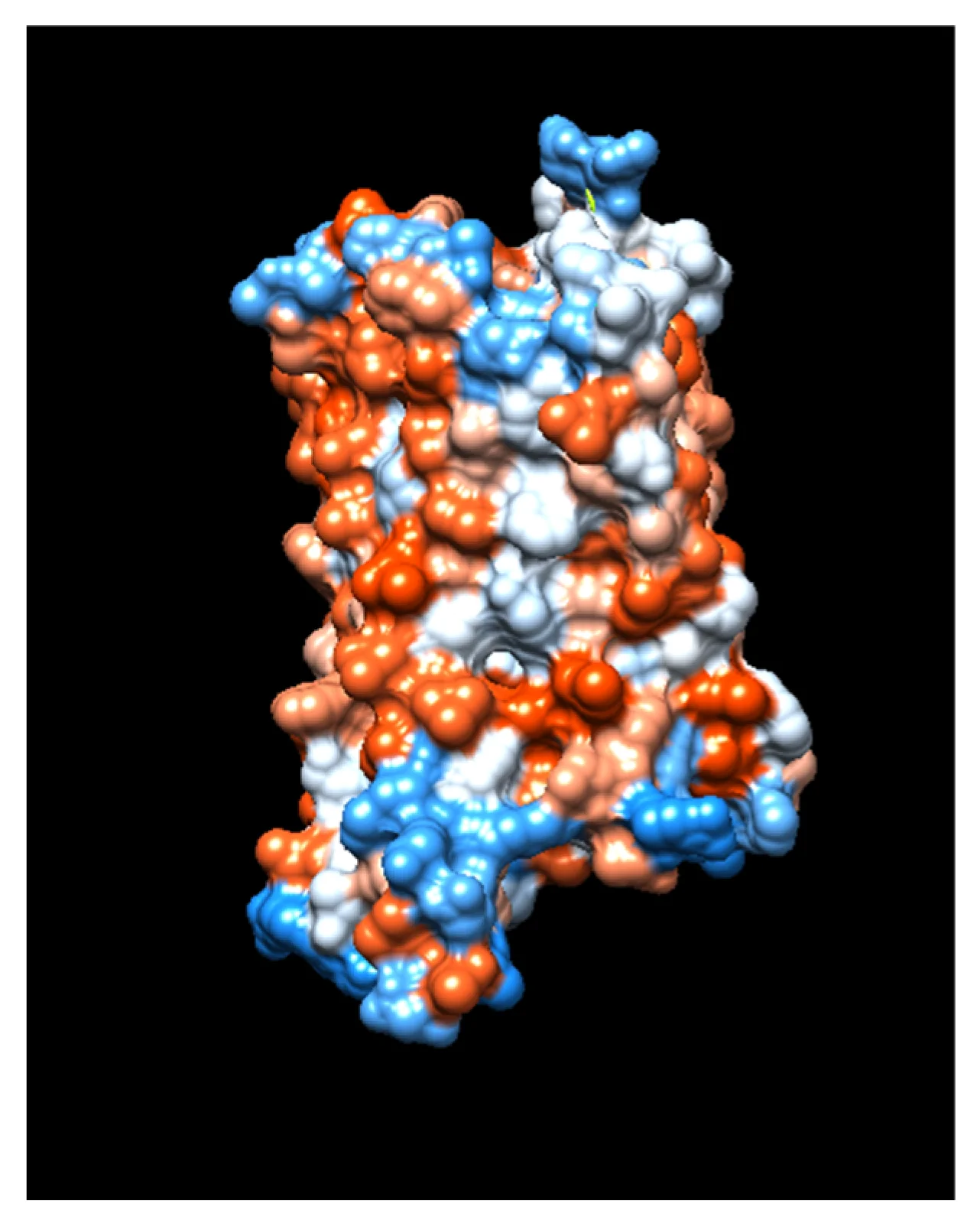
Coulombic electrostatic potential mapped onto the CPK surface representation of NK2R (chain B of PDB ID: 7XWO). Electrostatic potentials were calculated using the Coulombic Surface Coloring function implemented in UCSF Chimera. Blue regions represent positive electrostatic potential, red regions represent negative electrostatic potential, and white regions indicate near-neutral charge distribution. The internal cavity corresponds to the orthosteric ligand-binding pocket formed by the transmembrane helical bundle. The extracellular entrance of this cavity includes a region of near-neutral electrostatic potential that may facilitate accommodation of amphipathic ligands.

To conceptually illustrate the potential interface between paclitaxel and NK2R, the experimentally determined structure (PDB ID: 7XWO) was visualized in UCSF Chimera [27]. An electrostatic surface representation was generated using the Coulombic Surface Coloring function implemented in UCSF Chimera. This method calculates electrostatic potential based on atomic partial charges using Coulomb’s law under vacuum conditions and maps the resulting values onto the solvent-accessible (CPK) surface of the isolated NK2R receptor model. Figure 5 and Figure 6 were regenerated and updated to ensure consistency between structural visualization, electrostatic surface representation, and the revised methodological description of receptor preparation. Hydrophobic surface mapping revealed a continuous nonpolar pocket (residues 240–270) within the transmembrane helices that overlapped with the canonical neurokinin-binding groove. When overlaid with the paclitaxel molecular model, spatial complementarity was observed between the hydrophobic benzene-ester moieties of paclitaxel and the lipophilic cavity of the receptor, suggesting a feasible orientation for weak van der Waals or hydrophobic interactions.

The receptor displays the canonical seven-transmembrane α-helix topology of GPCRs, which is stabilized by multiple intramolecular hydrogen bonds within its helical core. Electrostatic surface mapping revealed predominantly neutral-to-hydrophilic regions on the extracellular surface, consistent with ligand recognition, and a hydrophobic transmembrane core that facilitates interaction with lipophilic ligands.

The receptor comprises seven transmembrane α-helices connected by extracellular and intracellular loops, a short N-terminal segment exposed to the extracellular environment, and a C-terminal cytoplasmic tail involved in G-protein coupling. Hydrogen bond mapping reveals multiple intramolecular interactions that stabilize the transmembrane core (Figure 5).

The analysis revealed predominantly neutral and hydrophilic regions on the extracellular surface, facilitating ligand recognition, whereas hydrophobic zones predominated in the transmembrane region. Electrostatic surface visualization of NK2R revealed distinct regions of positive and negative potential distributed across the receptor surface (Figure 6). The visualization of the hydrophobic surface reveals an amphipathic distribution, with hydrophilic (blue) residues on the extracellular side and hydrophobic (red) residues facing the lipid bilayer. The transmembrane helical bundle forms an internal cavity corresponding to the orthosteric ligand-binding pocket characteristic of class A GPCRs. This pocket provides a predominantly hydrophobic environment suitable for accommodating lipophilic ligands such as paclitaxel. These structural features—including the amphipathic distribution and the hydrophilic extracellular and hydrophobic transmembrane regions—are characteristic of GPCRs and are essential for dynamic ligand-receptor interactions.

We hypothesize a potential secondary interaction between paclitaxel and NK2R. Qualitative structural comparison supported the SwissTargetPrediction score of 0.32, suggesting moderate structural similarity to previously reported NK2R ligands. Electrostatic surface visualization (Figure 6) revealed a region of near-neutral electrostatic potential located within the extracellular entrance of the receptor binding pocket. This area appears as a predominantly white region in the Coulombic surface representation, indicating minimal net charge. Such a neutral microenvironment may facilitate the accommodation of large amphipathic molecules with limited net charge, such as paclitaxel. These observations provide a structural context for the proposed secondary interaction hypothesis, without implying high specificity of binding.

### 3.6. Integrated Computational Interpretation

The combined computational analyses suggest that paclitaxel possesses physicochemical characteristics compatible with transient accommodation within hydrophobic transmembrane receptor environments such as NK2R. Ligand-based target prediction, molecular docking, and molecular dynamics simulations collectively indicated a structurally plausible interaction model between paclitaxel and the NK2R binding cavity. The observed interaction pattern was dominated primarily by hydrophobic contacts and remained relatively stable throughout the molecular dynamics trajectories. These findings should be interpreted in the context of the study’s reliance exclusively on computational modeling approaches. SwissTargetPrediction provides similarity-based probabilistic predictions and does not establish receptor specificity or biological activity. Likewise, docking scores and MM-PBSA calculations provide theoretical estimates of structural compatibility rather than experimental measures of pharmacological binding. Consequently, the proposed NK2R–paclitaxel interaction should be considered a hypothesis-generating computational observation that may justify future experimental investigation.

### 3.7. Molecular Docking Analysis (Simulated Study)

**Figure 7:**
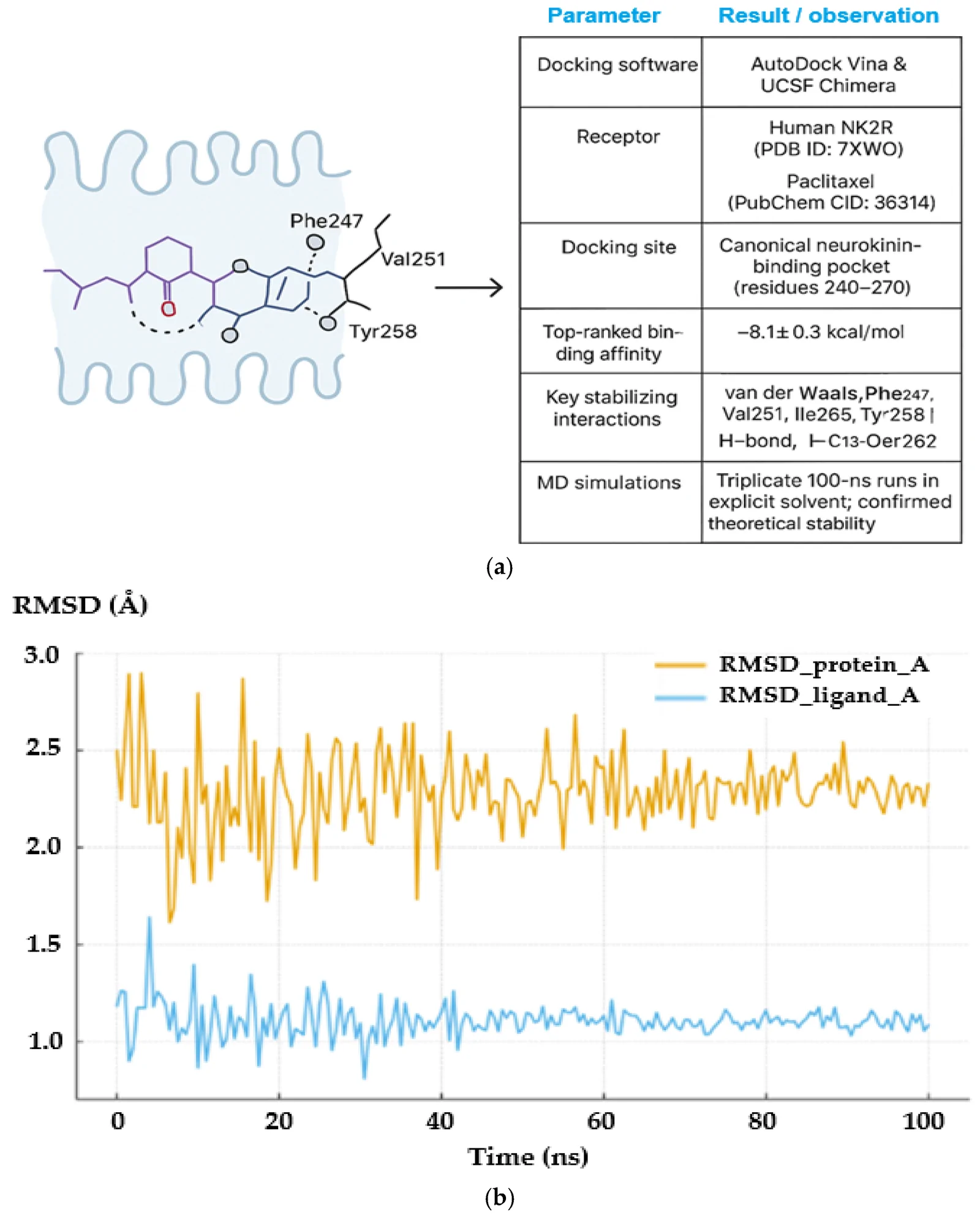

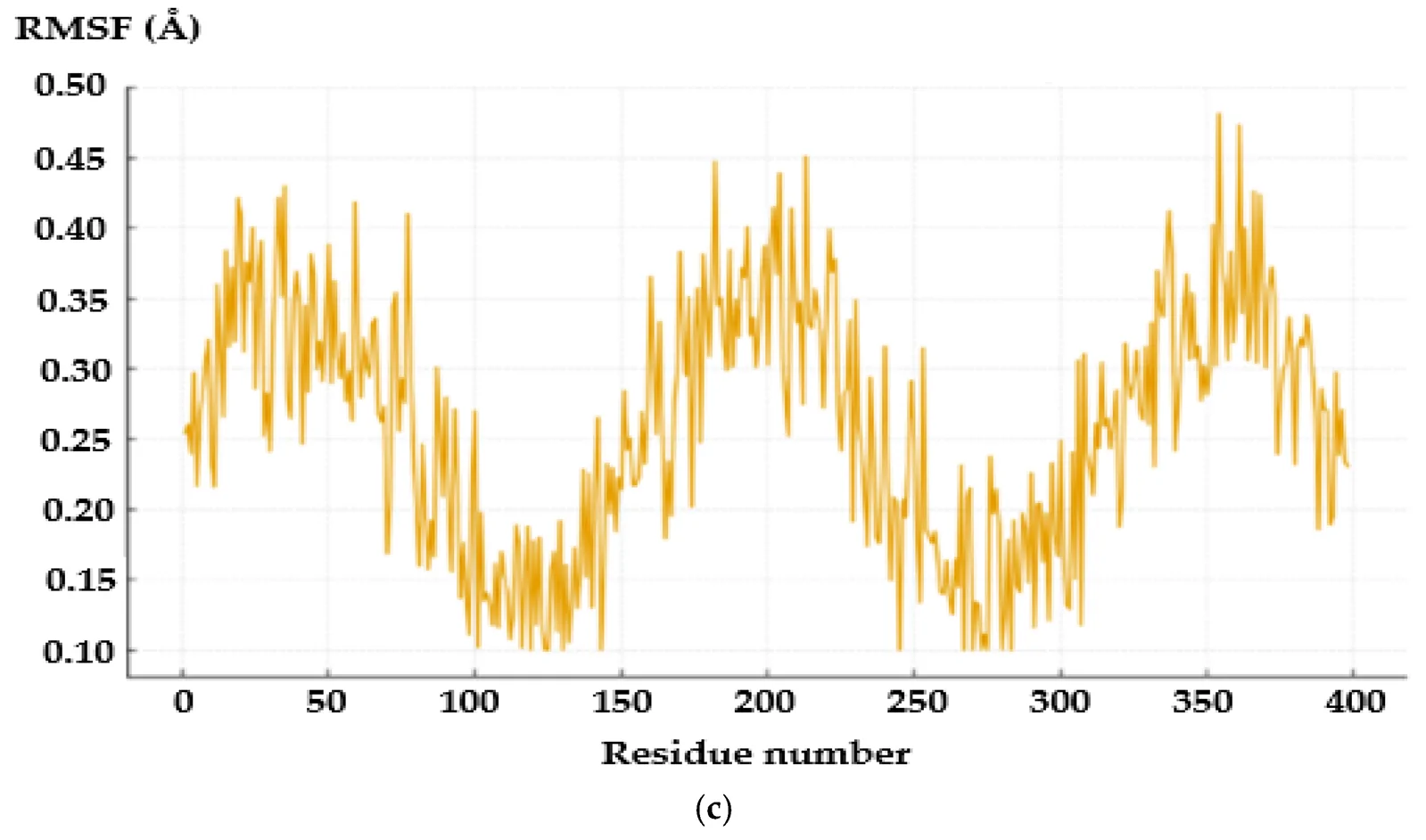
Docking and molecular dynamics analysis of the NK2R–paclitaxel complex. (**a**) Predicted docking pose of paclitaxel within the orthosteric binding pocket of NK2R, obtained from molecular docking calculations. The ligand is shown in stick representation within the transmembrane binding cavity of the receptor. (**b**) Root mean square deviation (RMSD) of the NK2R backbone atoms during the 100 ns molecular dynamics simulation, reflecting the structural stability of the receptor. (**c**) RMSD of paclitaxel heavy atoms relative to the initial docking conformation during the MD simulation, illustrating ligand stability within the binding pocket. RMSD values are reported in Å (Ångström). Curves represent the average trajectory behavior obtained from three independent 100 ns simulations.

The top-ranked docking pose of paclitaxel exhibited a Vina docking score of −8.1 ± 0.3 kcal·mol^−1^ for the canonical neurokinin-binding pocket (Figure 7a). This value corresponds to the docking scoring function used to rank predicted ligand poses and should not be interpreted as a true thermodynamic binding free energy. Docking scores are semi-empirical estimates that primarily serve to compare the relative stability of predicted binding conformations. The selected docked configuration served as the starting structure for subsequent molecular dynamics simulations and MM-PBSA analysis. The ligand adopted a consistent orientation, maintaining the same relative position and alignment of functional groups, within the hydrophobic transmembrane groove, which was stabilized by van der Waals contacts with residues Phe247, Val251, Ile255, and Tyr258, and a weak hydrogen bond between the C13 hydroxyl group of paclitaxel and Ser262 (Figure 7a).

The reproducibility of the predicted binding orientation was supported by the high structural similarity among the top-ranked docking poses obtained from repeated runs (RMSD < 1.5 Å). The selected docking pose therefore represents a representative binding conformation within the most consistent cluster of solutions predicted by the docking protocol. The RMSD plot (Figure 7b) shows that the ligand–protein complex remains stable over the simulation period, with fluctuations within an acceptable range, indicating structural convergence. RMSD of the protein backbone increased rapidly during the initial equilibration phase (0–20 ns), then plateaued at approximately 2.3 ± 0.4 Å, indicating overall global structural stabilization after equilibration. However, because RMSD is a global structural metric, this plateau should not be interpreted as evidence that the receptor remained exclusively in its initial active-like conformational state. In particular, subtle activation-state-related rearrangements may occur without producing large changes in the overall backbone RMSD. Therefore, the present RMSD analysis supports global structural stability of the receptor during the simulated trajectory but cannot by itself exclude a partial shift toward an inactive-like conformation. The ligand RMSD, calculated relative to the receptor-binding pocket, remained below 1.8 Å for the entire trajectory, with an average value of 1.1 Å. This observation is consistent with paclitaxel maintaining a stable orientation within the hydrophobic cavity of NK2R. The convergence of both RMSD trajectories after approximately 20 ns suggests that the complex reached a dynamic equilibrium and remained structurally stable over the remainder of the simulation. This convergence and observed structural stability support the hypothesis that paclitaxel forms an energetically favorable, non-covalent interaction with NK2R, primarily stabilized by hydrophobic contacts within the transmembrane domain (Figure 7b).

Our analysis of RMSF values was performed to examine the flexibility of individual residues within the NK2R–paclitaxel complex during the 100 ns simulation. Most residues exhibited low to moderate fluctuations, with average RMSF values ranging between 0.2 and 0.5 Å, indicating a generally stable transmembrane structure. The lowest mobility values were observed within the α-helical transmembrane segments, consistent with their structural rigidity and role in maintaining receptor conformation (Figure 7c). In contrast, higher RMSF values were detected in several extracellular and intracellular loop regions, reflecting the intrinsic flexibility of these segments and their involvement in ligand recognition and signal transduction (Figure 7c). The overall fluctuation pattern was typical of that observed in class A GPCRs, consistent with conformational stability of NK2R throughout the simulation while allowing limited local motions necessary for functional dynamics. The observed RMSF patterns and overall structural stability further support the dynamic stability of the NK2R–paclitaxel complex and suggest that paclitaxel binding does not induce significant conformational perturbations in the receptor’s backbone.

The selected docking pose served as the starting structure for molecular dynamics (MD) simulations aimed at evaluating the energetic stability of the complex in an explicit-solvent environment. To obtain a more reliable estimate of binding stability, binding free energy calculations were performed using the MM-PBSA method on the resulting MD trajectories. Although these analyses were conducted entirely in silico, the combined docking, molecular dynamics, and MM-PBSA results provide complementary structural and energetic estimates, supporting the proposed paclitaxel–NK2R interaction.

These analyses suggest an energetically plausible interaction between paclitaxel and NK2R, consistent with the receptor’s hydrophobic transmembrane environment without significant translation or positional shifts in the ligand during the MD simulations.

### 3.8. Triplicate Molecular Dynamics Simulations

All three simulated replicates showed comparable stability, with the complex maintaining its overall structural integrity throughout the simulation. The average RMSD of the protein backbone plateaued at 2.3 ± 0.4 Å after 20 ns. In contrast, the ligand RMSD relative to the binding pocket remained below 1.8 Å, indicating persistent occupancy of paclitaxel within the hydrophobic cavity (Table 3). The RMSF analysis revealed limited flexibility in transmembrane helices II–VI, whereas the extracellular loops exhibited higher mobility, consistent with typical GPCR behavior.

The calculated MM-PBSA binding free energy averaged −42.6 ± 3.7 kJ mol^−1^ (approximately −10.2 kcal mol^−1^) across the three independent 100 ns trajectories. This value provides an energetically favorable estimate based on molecular-mechanics and solvation contributions and is consistent with the overall structural stability observed during the simulations. However, because entropic contributions were not explicitly calculated, this value should not be interpreted as a complete absolute binding free energy or as a direct quantitative measure of pharmacological affinity. The omission of an unfavorable binding entropy contribution could make the calculated value more favorable than the corresponding entropy-corrected free energy (Table 3). Decomposition of the binding free energy revealed dominant van der Waals (−35 ± 2 kJ mol^−1^) and nonpolar solvation (−9 ± 1 kJ mol^−1^) contributions, consistent with the hydrophobic nature of the NK2R transmembrane cavity (Table 3).

The MM-PBSA binding free energy should not be directly compared with the AutoDock Vina docking score because the two quantities are derived from different computational frameworks. Whereas the docking score represents an empirical estimate obtained from a rigid-receptor docking calculation, the MM-PBSA value reflects the average binding free energy of the equilibrated protein–ligand complex sampled during molecular dynamics simulations. The slightly more favorable MM-PBSA value therefore likely reflects structural relaxation and stabilization of the complex under dynamic conditions.

Although these computational findings are exploratory, the complementary docking and molecular dynamics results indicate reasonable internal consistency between the static and dynamic models. The simulated complex remained conformationally stable throughout the 100-ns trajectories, with the ligand maintaining persistent occupancy in the binding pocket. Taken together, these results provide a theoretical energetic rationale for the predicted NK2R–paclitaxel interaction, reinforcing its plausibility as a secondary binding site.

The selected docking conformations were visually inspected to confirm preservation of paclitaxel stereochemistry and integrity of the strained ring system prior to molecular dynamics simulations.

## 4. Discussion

Paclitaxel, the active pharmaceutical ingredient in Abraxane, is a cornerstone agent in the treatment of breast cancer and other solid tumors due to its established cytotoxic mechanism targeting microtubule dynamics [7,8]. Beyond tubulin stabilization, growing evidence supports pleiotropic effects of paclitaxel across signaling pathways involved in tumor progression, angiogenesis, and immune regulation [9,16,28]. These features make paclitaxel a relevant candidate for systematic computational exploration of secondary molecular interactions.

The computational workflow integrates complementary analytical layers to characterize both ligand properties and target compatibility. SwissADME and SwissTargetPrediction were used for physicochemical and target-space profiling, while UCSF Chimera and GROMACS enabled structural refinement and energetic characterization. Together, these established platforms provide a reproducible framework for multi-level in silico assessment of drug–target interactions.

In the Abraxane formulation, paclitaxel is released from albumin-bound nanoparticles in a controlled manner that shapes its systemic distribution [12,29]. Pharmacokinetic data indicate that unbound paclitaxel reaches low nanomolar concentrations in plasma and tissues [30,31,41], with sustained release extending exposure time [42]. These conditions are consistent with the potential for interaction with membrane-associated targets, including GPCRs, particularly in tissue compartments with drug accumulation. Although circulating concentrations of unbound paclitaxel are generally low, receptor occupancy cannot be inferred directly from computational binding energies alone. In addition, the albumin-based formulation of Abraxane promotes heterogeneous tissue distribution and prolonged exposure, potentially leading to local drug concentrations that differ from plasma levels. Whether these concentrations are sufficient to produce pharmacologically relevant NK2R engagement remains unknown and will require experimental pharmacokinetic and pharmacodynamic investigations. Therefore, the present computational findings should be interpreted as supporting structural feasibility rather than demonstrating clinically relevant receptor modulation.

The physicochemical profile of paclitaxel reflects its known pharmacological behavior. The molecule exhibits high molecular weight, low gastrointestinal absorption, and limited blood–brain barrier permeability [29,43]. As a result, systemic distribution relies primarily on albumin-mediated transport pathways, including gp60–caveolin-1 transcytosis and SPARC-associated uptake mechanisms [32,33]. The low bioavailability score (0.17) and deviations from classical drug-likeness criteria are consistent with the nanoparticle-dependent pharmacokinetic profile of paclitaxel [43].

NK2R, a tachykinin receptor family member, regulates smooth muscle contraction, pain transmission, and inflammatory signaling via the phosphatidylinositol–calcium pathway [34,44]. In oncology, tachykinin signaling has been implicated in tumor proliferation, migration, and angiogenesis, with documented overexpression of NK1R and NK2R across multiple cancer types, including breast carcinoma [45,46,47,48,49,50].

Growing evidence supports the involvement of the Substance P/neurokinin-1 receptor (SP/NK1R) signaling axis in cancer progression, where it contributes to tumor growth, invasion, metastasis, and resistance to therapy. Consequently, NK1R has emerged as a promising therapeutic target across multiple malignancies, including pancreatic cancer, glioblastoma, osteosarcoma, and colorectal cancer, with preclinical studies demonstrating encouraging antitumor effects of NK1R antagonists such as aprepitant. These findings further support the rationale for investigating neurokinin receptor–associated pathways as potential targets in oncology and provide a broader biological context for the present computational analysis [51,52,53,54,55,56].

Within this context, NK2R represents a structurally and biologically relevant candidate within the predicted target space of paclitaxel. This finding extends previous reports linking tachykinin signaling with taxane pharmacology [57,58] by providing a quantitative and structure-based characterization of the interaction hypothesis through an integrated computational pipeline. NK2R was prioritized not only because of its predicted interaction probability but also because it fulfilled multiple predefined selection criteria, including biological relevance to breast cancer, documented involvement in tachykinin signaling, and the availability of a high-resolution experimentally determined human receptor structure suitable for structure-based modeling. Other predicted GPCR targets, such as dopamine D2 and β-adrenergic receptors, remain of potential interest and may warrant investigation in future computational and experimental studies.

SwissTargetPrediction assigned paclitaxel a moderate probability score for NK2R (p = 0.32), consistent with its structurally complex and non-classical pharmacophore profile. Molecular docking and triplicate molecular dynamics simulations are consistent with a structurally stable binding mode over the simulated timescale, while the overall interaction is interpreted as potentially transient. The agreement between methods indicates robust convergence across static and dynamic energy models.

Structurally, NK2R displays a predominantly hydrophobic transmembrane core with polar extracellular regions [58,59,60], forming an environment compatible with amphipathic ligands such as paclitaxel. The conserved architecture of NK2R across mammalian species [61,62] further supports the functional relevance of its ligand-binding cavity for diverse chemical scaffolds.

The predominance of hydrophobic contacts observed in the predicted binding mode is consistent with the highly lipophilic character of paclitaxel. However, such interactions alone do not establish receptor specificity, and non-specific partitioning into hydrophobic regions of membrane proteins cannot be excluded. Consequently, the present computational results should be interpreted as supporting the structural plausibility of an NK2R–paclitaxel association rather than demonstrating selective receptor recognition. Experimental binding and functional studies will be required to determine whether the predicted interaction reflects specific receptor engagement or a more general membrane-associated binding phenomenon.

From a mechanistic perspective, the predicted interaction suggests a potential link between paclitaxel exposure and modulation of tachykinin-associated signaling networks in cancer. This aligns with established roles of neurokinin receptors in tumor biology. The current computational results support a structurally plausible interaction hypothesis, while functional consequences remain to be explored experimentally. The analysis is based on a representative receptor conformation and captures a structurally relevant state of NK2R suitable for small-molecule engagement assessment.

The NK2R structure (PDB ID: 7XWO) represents an active-state receptor complexed with a peptide agonist and G-protein, without a co-crystallized small-molecule antagonist. In this context, docking results provide a structural feasibility map of the binding region, complemented by molecular dynamics simulations that evaluate stability under explicit solvent conditions.

Taken together, the docking and MM-PBSA analyses provided complementary information on the predicted interaction, with docking describing the preferred binding configuration and MM-PBSA providing an energetic estimate based on molecular mechanics and solvation terms. Because no comparative analysis with experimentally characterized NK2R agonists or antagonists was performed, the present study cannot determine whether paclitaxel occupies the canonical NK2R binding site or an alternative receptor cavity. Accordingly, the proposed binding mode should be interpreted as a structurally plausible hypothesis that warrants further investigation through comparative structural studies and experimental validation.

Although residue-level interaction mapping was not the focus of this study, future work incorporating detailed contact profiling (hydrogen bonding, hydrophobic interactions, aromatic stacking) will further refine the binding model.

The integrated computational framework (SwissADME, SwissTargetPrediction, UniProt, ProtParam, Chimera, GROMACS) enables a systematic characterization of paclitaxel from both pharmacokinetic and target-structure perspectives. This approach supports efficient prioritization of biologically relevant hypotheses in early-stage drug interaction analysis.

Within the broader context of breast cancer research, multi-stage computational workflows combining docking, molecular dynamics, and free energy calculations are widely used to characterize protein–ligand systems. Studies such as Shoaib et al. [63] on CK2 inhibition in triple-negative breast cancer, CDK1/PARP1 dual-target inhibitor design [64], and quantum chemical analyses of aromatase inhibitors [65] illustrate the established role of integrated in silico methodologies in identifying and validating therapeutic hypotheses. Tiwari and Khan characterized the Bcl-2 gene and apoptotic pathways in the MCF-7 breast cancer cell line, identifying apoptotic regulators as key therapeutic targets. Although experimental, their findings support the relevance of Bcl-2-mediated apoptosis as a target for subsequent in silico studies using molecular docking and dynamics simulations [66].

This study identifies NK2R as a structurally supported candidate within the predicted target space of paclitaxel and provides a coherent computational framework linking pharmacokinetic properties to GPCR-level interaction potential. The results establish a testable hypothesis for paclitaxel–NK2R engagement and provide a structured basis for future experimental validation.

## 5. Limitations

Several methodological limitations should be considered when interpreting the results of this study. The analysis is based entirely on computational approaches; therefore, it characterizes a structural and energetic interaction hypothesis rather than experimentally observed binding. Experimental validation using receptor-binding assays, cellular functional studies, and pharmacological characterization remains necessary to confirm biological relevance.

The molecular docking analysis was conducted using a single experimentally resolved active-state structure of NK2R (PDB ID: 7XWO). Because no experimentally determined ligand-free or inactive-state human NK2R structure was available for direct comparative docking, receptor conformational variability could not be explicitly sampled. This is particularly relevant because paclitaxel is not proposed as a canonical NK2R agonist, and its potential interaction may therefore be dependent on receptor conformational state. Accordingly, the present results should not be interpreted as evidence of state-independent paclitaxel binding. Comparative docking and molecular dynamics simulations using experimentally resolved active- and inactive-state NK2R conformations would be valuable for determining whether the predicted binding mode and energetic profile are state-dependent. Furthermore, the backbone RMSD plateau observed during molecular dynamics is consistent with global structural stabilization but does not by itself distinguish between active-like and inactive-like receptor conformations. Subtle conformational rearrangements associated with GPCR activation or deactivation may occur without producing substantial changes in global RMSD. In addition, the docking protocol was implemented as an exploratory structural assessment rather than a classical virtual screening workflow. Because the available NK2R structure contains a peptide agonist rather than a small-molecule ligand, standard redocking-based validation could not be performed. Redocking of the co-crystallized peptide was not considered appropriate because peptide agonists and structurally complex small molecules such as paclitaxel differ substantially in their binding mechanisms and interaction profiles. Consequently, assessment of the predicted binding pose relied on its structural stability during molecular dynamics simulations and on the consistency of the MM-PBSA binding free-energy estimates rather than on peptide redocking. Accordingly, docking scores should be interpreted as indicators of structural compatibility and binding plausibility rather than quantitative estimates of pharmacological potency.

Molecular dynamics simulations and MM-PBSA calculations were used to complement docking results by assessing complex stability under dynamic conditions. However, these approaches rely on force-field approximations and simplified energetic decompositions. Entropic contributions were not explicitly computed in the MM-PBSA analysis. This is relevant for paclitaxel because its large and conformationally complex structure may incur an unfavorable entropy contribution upon binding that is not captured by the present calculations. Consequently, the reported MM-PBSA value should be interpreted as an energetic estimate based on molecular mechanics and solvation terms rather than as a fully entropy-corrected absolute binding free energy. Furthermore, convergence assessment was based primarily on RMSD analysis and the reproducibility of three independent simulation trajectories. More advanced analyses, such as principal component analysis (PCA), cluster analysis, or essential dynamics, were beyond the scope of the present study but could provide additional insight into the conformational landscape of the NK2R–paclitaxel complex in future investigations.

The omission of an explicit lipid bilayer was an intentional simplification consistent with the exploratory nature of the present study. The primary objective of the molecular dynamics simulations was to evaluate the intrinsic stability of the predicted NK2R–paclitaxel complex after docking rather than to reproduce the complete physiological environment of a membrane-embedded GPCR. Importantly, the membrane environment may influence the accessibility and geometry of the predominantly hydrophobic transmembrane cavity through lipid–protein interactions and receptor conformational changes. This may be particularly relevant for paclitaxel because of its large and lipophilic molecular scaffold and its potential for membrane-associated accommodation. Therefore, the present simulations cannot establish whether the hydrophobic residues identified in the predicted binding site would be more or less accessible under membrane-embedded conditions.

An additional constraint arises from the structural characteristics of paclitaxel. Its large, rigid, and highly substituted scaffold differs substantially from canonical NK2R ligands, which suggests that any interaction may be more consistent with transient or membrane-assisted binding than with stable orthosteric receptor engagement. This structural context should be considered when interpreting predicted binding modes.

In addition, the docking protocol preserved the rigid polycyclic taxane core while allowing rotation of non-ring rotatable bonds. Although this approach reflects the intrinsic structural characteristics of paclitaxel, it may restrict the exploration of alternative ligand conformations and binding poses that could become accessible under different receptor conformations or longer simulation times.

Finally, the study focused exclusively on paclitaxel as the active compound of Abraxane and did not explicitly model the albumin-bound nanoparticle formulation. Consequently, formulation-dependent processes such as albumin-mediated transport, SPARC interactions, and local release kinetics were not included in the structural simulations and were considered only at a conceptual level.

The present analysis was restricted to the native paclitaxel stereoisomer and did not explicitly consider epimeric forms or degradation products. No separate simulations were performed for alternative stereoisomers or chemically degraded species. Such species may exhibit different conformational and binding properties and could be investigated in future comparative computational studies.

## 6. Future Perspectives and Clinical Implications

The computational findings presented in this study establish a structural framework for exploring potential receptor-level interactions of paclitaxel beyond its canonical microtubule-targeting mechanism. The NK2R–paclitaxel interaction identified herein provides a clear basis for further experimental and computational investigation.

Future experimental work should evaluate the predicted interaction using complementary biochemical and cellular approaches. Receptor-binding assays, calcium flux measurements, downstream signaling analyses, and functional assays in NK2R-expressing breast cancer models would directly assess whether the observed structural compatibility translates into measurable pharmacological effects.

From a computational standpoint, further refinement of GPCR modeling is warranted through ensemble docking strategies, enhanced-sampling molecular dynamics simulations, and membrane-explicit systems that more accurately reproduce the native lipid environment of NK2R. The use of both active and inactive receptor conformations would further clarify state-dependent binding behavior and ligand accessibility.

Comparative analyses with experimentally evaluated NK2R agonists and antagonists represent another relevant direction. Such comparisons, based on binding poses, interaction fingerprints, and energetic profiles, would help delineate whether paclitaxel engages canonical orthosteric sites or interacts preferentially through transient hydrophobic regions within the transmembrane domain.

Given that this study focused on free paclitaxel, future investigations should incorporate formulation-dependent factors associated with Abraxane. Explicit modeling of albumin–paclitaxel complexes, along with membrane-associated delivery mechanisms, may provide additional insight into how nanoparticle-based pharmacokinetics influence receptor accessibility and local drug concentration in tumor environments.

More broadly, the integrated workflow developed here provides a framework that could be applied to other amphipathic cytotoxic agents. Comparative studies of related taxanes and other cytotoxic compounds will be needed to assess its reproducibility and broader applicability. Taken together, these approaches provide a structured pathway for linking computational predictions with targeted experimental validation, with the aim of clarifying whether GPCR-associated interactions contribute to the broader pharmacodynamic profile of taxane-based therapies and whether such mechanisms may hold relevance for future therapeutic or biomarker development.

## 7. Conclusions

The present study investigated the potential interaction between paclitaxel and the NK2R using an integrated computational workflow combining physicochemical profiling, target prediction, molecular docking, and molecular dynamics simulations. The results indicate that paclitaxel exhibits structural and physicochemical features compatible with transient accommodation within the hydrophobic transmembrane cavity of NK2R. Docking analyses revealed a consistent binding mode, primarily stabilized by hydrophobic interactions, while molecular dynamics simulations indicated sustained structural stability of the predicted complex. MM-PBSA calculations provided an additional energetic estimate consistent with the proposed interaction.

Collectively, these findings support a structurally plausible NK2R–paclitaxel interaction within a computational framework that links ligand physicochemical properties with receptor-level binding behavior. The results extend the known pharmacological profile of paclitaxel by highlighting a potential secondary interaction space within GPCR environments, consistent with its amphipathic and membrane-accessible characteristics.

The strength of this work lies in its integrated strategy, which combines pharmacokinetic descriptors, target prediction, and structure-based simulations to systematically evaluate unconventional interaction hypotheses for a clinically established cytotoxic agent. This approach provides a reproducible computational pipeline for prioritizing secondary receptor interactions with potential relevance in oncology.

Further investigation of the NK2R–paclitaxel interaction using experimental receptor-binding studies, functional cellular assays, and membrane-explicit simulations will clarify its biological significance and potential relevance in tumor signaling contexts. Such studies will help define whether GPCR-associated interactions contribute to the broader pharmacodynamic landscape of paclitaxel-containing therapies.

## Figures and Tables

**Table 1:** Key physicochemical descriptors of paclitaxel (PubChem CID: 36314).

Descriptor	Value	Interpretation
Molecular weight (g/mol)	853.9	High; exceeds Lipinski threshold
logP	2.5	Moderate lipophilicity
TPSA (Å^2^)	221	Suggests low membrane permeability
Rotatable bonds	14	Indicates structural flexibility
HBD/HBA	4/14	Multiple hydrogen bonding sites

**Table 2:** Predicted pharmacokinetic properties and drug-likeness assessment (SwissADME).

Property	Predicted Outcome	Quantitative Value/Note
GI absorption	Low	—
BBB permeability	Negative	—
P-gp substrate	Yes	Supported
CYP inhibition (1A2, 2C19, 2C9, 2D6, 3A4)	None	—
Bioavailability score	0.17	Low
Compliance with Lipinski/Veber/Ghose/Egan/Muegge rules	No	Violations in MW, TPSA, and flexibility

**Table 3:** Simulated dynamics and binding free energy of paclitaxel at NK2R (Legend: blue—RMSD/structural stability, green—RMSF/flexibility, red—binding free energy/docking score, and black—proportional graphical bar illustrating the relative magnitude of the corresponding numerical values).

Parameter	Result	Mini-Graph (Proportional)	Notes
Protein backbone RMSD	2.3 ± 0.4 Å	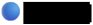	Plateau after ~20 ns indicates structural stability
Ligand RMSD (relative to pocket)	<1.8 Å	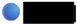	Paclitaxel remains stably in the hydrophobic cavity
Binding free energy (MM-PBSA)	−42.6 ± 3.7 kJ/mol (~−10.2 kcal/mol)	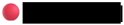	Average over 3 replicates, consistent with docking
Docking score	−8.1 kcal/mol	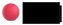	Supports consistency between static and dynamic models
van der Waals contribution	−35 ± 2 kJ/mol	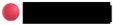	Dominant hydrophobic interactions
Non-polar solvation	−9 ± 1 kJ/mol	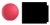	Minor hydrophobic contribution
RMSF (flexibility)	Low in helices II–VI; higher in extracellular loops	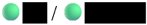	Typical GPCR behavior
Complex structural stability	Stable	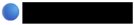	Ligand remains in pocket, supporting theoretical interaction

## Data Availability

All analyzed data are included in this article. Raw data can be obtained upon request.

## Abbreviations

The following abbreviations are used in this manuscript:

ADME	Absorption, Distribution, Metabolism, and Excretion
BBB	Blood–Brain Barrier
CID	Compound Identifier (PubChem Compound ID)
CYP	Cytochrome P450 (drug-metabolizing enzyme family)
GI	gastrointestinal
GPCR	G-Protein Coupled Receptor
GRAVY	Grand Average of Hydropathy (measure of protein hydrophobicity)
HBA	Hydrogen Bond Acceptors
HBD	Hydrogen Bond Donors
logP	logarithm of the partition coefficient
MM-GBSA	Molecular Mechanics Generalized Born Surface Area
MM-PBSA	Molecular mechanics for the internal energy with Poisson-Boltzmann and surface area (PBSA)
MW	Molecular Weight
NK1R	Neurokinin-1 Receptor
NK2R	Neurokinin-2 Receptor (tachykinin receptor 2)
NKRs	Neurokinin Receptors (family including NK1R, NK2R, NK3R)
PDB	Protein Data Bank
RMSD	Root mean square deviation
RMSF	Root mean square fluctuation
pI	Isoelectric Point
P-gp	P-glycoprotein (efflux transporter)
ProtParam/ProtScale	Bioinformatic tools for protein physicochemical analysis
SPARC	Secreted Protein Acidic and Rich in Cysteine (tumor-associated albumin receptor)
SwissADME	Swiss platform for ADME and drug-likeness prediction
SwissTargetPrediction	Computational tool predicting potential protein targets for bioactive molecules
TPSA	Topological Polar Surface Area
UniProt	Universal Protein Resource (protein sequence and annotation database)
Veber rule	Drug-likeness rule based on rotatable bonds and polar surface area
gp60	Albumin-binding endothelial glycoprotein 60
